# Problematic Shopping Behavior: An Item Response Theory Examination of the Seven-Item Bergen Shopping Addiction Scale

**DOI:** 10.1007/s11469-022-00844-8

**Published:** 2022-06-20

**Authors:** Daniel Zarate, Lana Fullwood, Maria Prokofieva, Mark D. Griffiths, Vasileios Stavropoulos

**Affiliations:** 1 Institute for Health and Sport, Victoria University, 70/104 Ballarat Road, Footscray, VIC 3011 Australia; 2 Victoria University, Footscray, Australia; 3 International Gaming Research Unit, Psychology Department, Nottingham Trent University, Nottingham, UK; 4 University of Athens, Athens, Greece

**Keywords:** Problematic shopping behavior; Shopping addiction; Bergen Shopping Addiction Scale; Item response theory; Differential item functioning

## Abstract

There has been an increasing amount of research examining problematic shopping behavior (PSB), often referred to in the psychological literature as “compulsive buying” or “shopping addiction.” A popular scale for assessing the risk of PSB is the seven-item Bergen Shopping Addiction Scale (BSAS). To expand our knowledge of the psychometric properties of this instrument, the present study employed Item Response Theory (IRT) and differential item functioning analyses (DIF) while concurrently attempting to determine a preliminary cut-off point. A relatively large community sample completed the BSAS online (*N* = 968, *M*_age_ = 29.5 years, *SD*_age_ = 9.36, 32.5% women). IRT analyses showed differences regarding the BSAS items’ discrimination, difficulty, and precision, with a raw score exceeding 23 (out of 28) indicating a higher risk of shopping addiction. Finally, while most BSAS items operated equally among males and females, Item 2 (*mood modification*) required a higher level of shopping addiction behaviors to be endorsed by males. The BSAS functions as a reliable assessment of the risk of shopping addiction, particularly between average and high levels of the trait. Clinical implications are discussed in light of these findings.

Scholars have investigated individuals’ “pathological propensity to buy,” suggesting the possibility of problematic shopping behavior (PSB; Aboujaoude, 2011; Aboujaoude, 2014; Andreassen et al., 2015; Georgiadou et al., 2021; Kyrios et al., 2018; Moulding et al., 2017 Müller et al., 2019; Müller et al., 2021a, 2021b; Rahman et al., 2018; Rigby, 2011; Uzarska et al., 2021). Many terms have been used to describe PSB (e.g., “compulsive buying,” “compulsive spending,” “shopping addiction,” “shopaholism,” “problematic shopping”) suggesting that such problematic behaviors  are associated with an inability to regulate emotions and/or excessive impulsivity (Christenson et al., 1994). However, recent research suggests that much like psychoactive substance addictions, PSB, and other problematic behaviors are best understood from an addiction perspective, given that internal factors (e.g., distress) and external factors (e.g., environmental cues) precipitate cue reactivity providing the basis for craving and anticipation of rewards (Gomez et al., 2022; Starcke et al., 2018).

Previous research has identified negative consequences associated with PSB (e.g., financial solvency, compromised social relationships, psychological distress), suggesting the recognition of shopping addiction as a distinct behavioral addiction in psychopathology classification manuals such as the DSM-5 or ICD-11 (Andreassen et al., 2018; APA, 2013; Dittmar, 2005; Griffiths, 1996, 2017, 2018; Hartston, 2012; Uzarska et al., 2021; WHO, 2019; Zarate et al., 2022; Zhao et al., 2017). However, concerns have also been raised regarding the potential risk of over-pathologizing common behaviors (e.g., work, exercise, sex), suggesting that problematic behaviors are likely to manifest when individuals engage in subjectively enjoyable activities (Kardefelt-Winther et al., 2017; Niedermoser et al., 2021). Given these concerns and the relatively recent conceptualization of behavioral addictions, it is important to evaluate *which symptoms* (if any) may be problematic or indicative of impaired wellbeing due to problematic shopping, and therefore providing conceptual clarification and empirical validity to PSB.

The lack of consistency surrounding the recognition of PSB as a formal diagnosis has raised questions concerning prevalence rates and individual differences (Andreassen et al., 2018; Georgiadou et al., 2021; Granero et al., 2016; Otero-Lopez et al., 2021; Potenza, 2014; Uzarska et al., 2021). For example, the reported prevalence of PSB has ranged between 4.9 and 16.2% (Black, 2001; Dittmar, 2005; Duroy et al., 2014; Maraz et al., 2016), with a hypothesized ascending trend due to consumerism and the recent COVID-19 pandemic (among other factors; Georgiadou et al., 2021; Granero et al., 2016; Niedermoser et al., 2021). Additionally, studies investigating gender differences in PSB have reported mixed findings, with some showing higher prevalence among females (Dittmar, 2005; Maraz et al., 2016; Otero-Lopez & Villardefrancos, 2014) and others reporting no gender differences (Jiang & Shi, 2016; Müller et al., 2010). These observed discrepancies could be partially attributed to the lack of solid psychometric understanding of the instruments used to assess PSB (Georgiadou et al., 2021).

Past research has employed multiple psychometric instruments encompassing a variety of definitions/conceptualizations of PSB (e.g., Compulsive Buying Measurement Scale, Valence et al., 1988; Online Shopping Addiction Scale, Zhao et al., 2017; Compulsive Online Shopping Scale, Manchiraju et al., 2017). Within this broader context of measurement inconsistencies and dearth of solid psychometric findings, the seven-item Bergen Shopping Addiction Scale (BSAS; Andreassen et al., 2015) has been used more consistently based on its versatility (i.e., online shopping and in-person shopping), promising psychometric performance, and sound theoretical approach (i.e., components model of addiction; Griffiths, 1996, 2005; Kaur et al., 2019; Tanoto & Evelyn, 2019; Uzarska et al., 2019, 2021; Zhao et al., 2017). It has been proposed that PSB includes seven core symptoms, comprising (i) excessive preoccupation with shopping (*salience*), (ii) shopping to change mood state (*mood modification*), (iii) inability to fulfill daily obligations due to shopping (*conflict*), (iv) increased amount of shopping over time to obtain satisfaction (*tolerance*), (v) return to excessive shopping after a period of controlled shopping (*relapse*), (vi) irritability and frustration in the absence of shopping (*withdrawal*), (vii) and impaired wellbeing due to excessive shopping (*problems*; Andreassen et al., 2015; Griffiths, 2005). Despite these advantages in using the BSAS, to the best of the present authors’ knowledge, there is limited evidence evaluating the scale at the item level employing advanced approaches such as item response theory (IRT). Such work would add clarity to the assessment of PSB and the appropriate estimation of its prevalence rates.

## Item Response Theory

It has been proposed that IRT outperforms classical test theory (CTT) approaches due to its ability to (i) assess relationships between item(s) and constructs, and therefore (ii) produce generalizable and sample independent results (Hambleton et al., 2010; Kircaburun et al., 2020). More specifically, IRT uses a logit function and logistic parameters (discrimination, *α*; difficulty, *β*; and pseudo-guessing, *c*) to assess item behavior at different levels of a latent trait *θ* (Embretson & Reise, 2013). In IRT, *α* evaluates how well an item “discriminates” between different *θ* levels (PSB), *β* examines the probability of endorsing an item at different *θ* levels, and *c* represents the probability of guessing “the correct response” to an item (De Ayala, 2008). Accordingly, IRT models can be estimated based on research needs, including “Rasch” models assuming equality constraints on α (Mellenbergh, 1994) or the graded response model assuming different α across items (GRM; Samejima, 1996). Although other models could be estimated (e.g., generalized partial credit, GPC; Muraki, 1992; nominal model for nominal and ordinal responses), the present study focuses on the Rasch and GRM due to its suitability for ordered polytomous items (Gomez et al., 2019; Marmara et al., 2021; Zarate et al., 2021).

Additionally, IRT provides three attractive features. Firstly, it can produce conditional precision indices (i.e., increased information produces lower standard errors increasing precision) to determine the reliability of a given instrument at different *θ* levels (Culpepper, 2013; Thomas et al., 2018). Secondly, it enables the estimation of prevalence rates via the employment of Summed Scores Expected a Posteriori (SSEAP [θ|x]) based on participants’ response patterns (i.e., raw scores ± 2 SD beyond the mean; Cai et al., 2011; Thissen, 1995). Thirdly, it can provide differential item functioning (DIF) statistics to investigate the equivalence of psychometric properties across groups (e.g., males and females; Meade & Wright, 2012).

## The Present Study

The present study adds to the extant literature by (i) investigating the psychometric properties of the BSAS including items’ discrimination (*α*) and difficulty (*β*), (ii) proposing an optimal raw cut-off score, and (iii) DIF statistics at the item level across males and females. These considerations are important since they may help identify items to be prioritized in clinical assessments based on the severity of the different PSB presentations. Additionally, they may enhance clarity considering the PSB prevalence rates and gender differences.

## Method

### Participants

The initial sample comprised 1097 English-speaking individuals from the general community. However, 129 responses were removed due to being invalid (e.g., spam, incomplete responses). Therefore, a final sample of 968 individuals aged between 18 and 64 years participated (*M*_age_ = 29.5 years, *SD* = 9.36; 315 females, 32.5%). The sample used in the present study exceed the suggested minimum sample size for IRT analysis (*N* items*15; 7 × 15 = 105; Sahin & Anil, 2017). Table 1 provides descriptive statistics, and Supplementary Table 1 provides demographic statistics. Gender groups showed homogeneity of variance (Levene’s *F* = 1.306, *p* = 0.254) and females scored significantly higher on the BSAS than males (*t* [895] = 3.949, *p* < *0.0*01).

**Table 1:** Addictive behaviors descriptive statistics (*N* = 968)

Items	Component	Males (*n* = 622)	Females (*n* = *315*)	Non-binary (*n* = 31)
1. I think about shopping/buying things all the time	Salience	2.44 (1.23)	2.81 (1.26)	2.42 (1.46)
2. I shop/buy in order to change my mood	Mood modification	2.35 (1.28)	2.94 (1.30)	2.81 (1.49)
3. I shop/buy so much that it negatively affects my daily obligations	Conflict	1.44 (0.81)	1.58 (0.87)	1.35 (0.75)
4. I feel I have to shop/buy more and more to obtain the same satisfaction as before	Tolerance	1.64 (0.99)	1.71 (0.99)	1.65 (1.11)
5. I have decided to shop/buy less, but have not been able to do so	Relapse	1.71 (0.99)	2.01 (1.13)	1.97 (1.25)
6. I feel bad if I for some reason am prevented from shopping/buying things	Withdrawal	1.81 (1.09)	1.96 (1.10)	1.68 (1.01)
7. I shop/buy so much that it has impaired my well-being	Presenting problems	1.46 (0.86)	1.50 (0.87)	1.55 (0.99)

*N* = sample size; standard deviation between parentheses; non-binary participants are those who did not identify as males or females

### Instrument

*Bergen Shopping Addiction Scale (BSAS):* The BSAS (Andreassen et al., 2015) assesses the risk of shopping addiction using seven items rated on a five-point Likert scale ranging from 0 (*strongly disagree*) to 4 (*strongly agree*). Originally item scores ranged from 1 (strongly disagree) - 5 (strongly agree) and have been converted to start from 0 to serve the present IRT analyses’ purposes. Each item relates to an element of the “components model of addiction” including salience, mood modification, tolerance, withdrawal symptoms, conflict, relapse, and presenting problems (Griffiths, 2005; Kim & Hodgins, 2018). Examples of items include *“I think about shopping/buying things all the time.”* Total possible scores range from 0–28, with higher scores indicating a higher risk of shopping addiction. The scale’s internal reliability in the present study was excellent (Cronbach’s *α* = 0.88, McDonald’s *ω* = 0.88).

### Procedure

The study was advertised via email (on the Victoria University student platform) and social media (*Twitter, Reddit, Facebook, Instagram*) after obtaining approval from the research team’s university Ethics Committee. Individuals over 18 years were eligible to participate and invited to complete an online survey including demographic questions and the BSAS. A Plain Language Information Statement was available upon accessing the link to ensure participant eligibility criteria were met (i.e., being adults), obtain informed consent, and ensure participation was voluntary. Data were collected between November 2020 and January 2021.

### Statistical Analyses

Statistical analyses followed a sequential process. First, IRT models were estimated with IRT-PRO (Cai et al., 2011). Model fit was concurrently determined by: (i) traditional fit indices (*χ*^2Loglikelihood^); (ii) marginal likelihood information statistics *M*_2_ (one and two-way marginal tables to correct for potentially sparse information); (iii) RMSEA (< 0.06 = sufficient fit; Hu & Bentler, 1999; Gomez et al., 2021a); and (iv) estimation of error prediction based on Akaike information criterion (AIC; Akaike, 1974) and Bayesian information criterion (BIC; Schwarz, 1978). Given the potential sensitivity of *M*_2_ to large samples (*N* > 900), emphasis was placed on RMSEA to assess model fit (De Ayala, 2008). Subsequently, the best fitting model was determined based on Δ*χ*^2Loglikelihood^ (Gomez et al., 2021b). Secondly, following past recommendations (Zarate et al., 2021), DIF statistics using Wald tests were obtained for all items with *p* < 0.05 as indication of non-invariance. Subsequently, to avoid increasing type 1 error, invariant items were anchored, and only non-invariant items were assessed. Thirdly, the conversion of the BSAS raw scores into addictive shopping risk levels was conducted based on SSEAP [*θ*|*x*] to classify participants exceeding ± 2SD as high risk (Cai et al., 2011; Embretson & Reise, 2013).

## Results

**Fig. 1:**
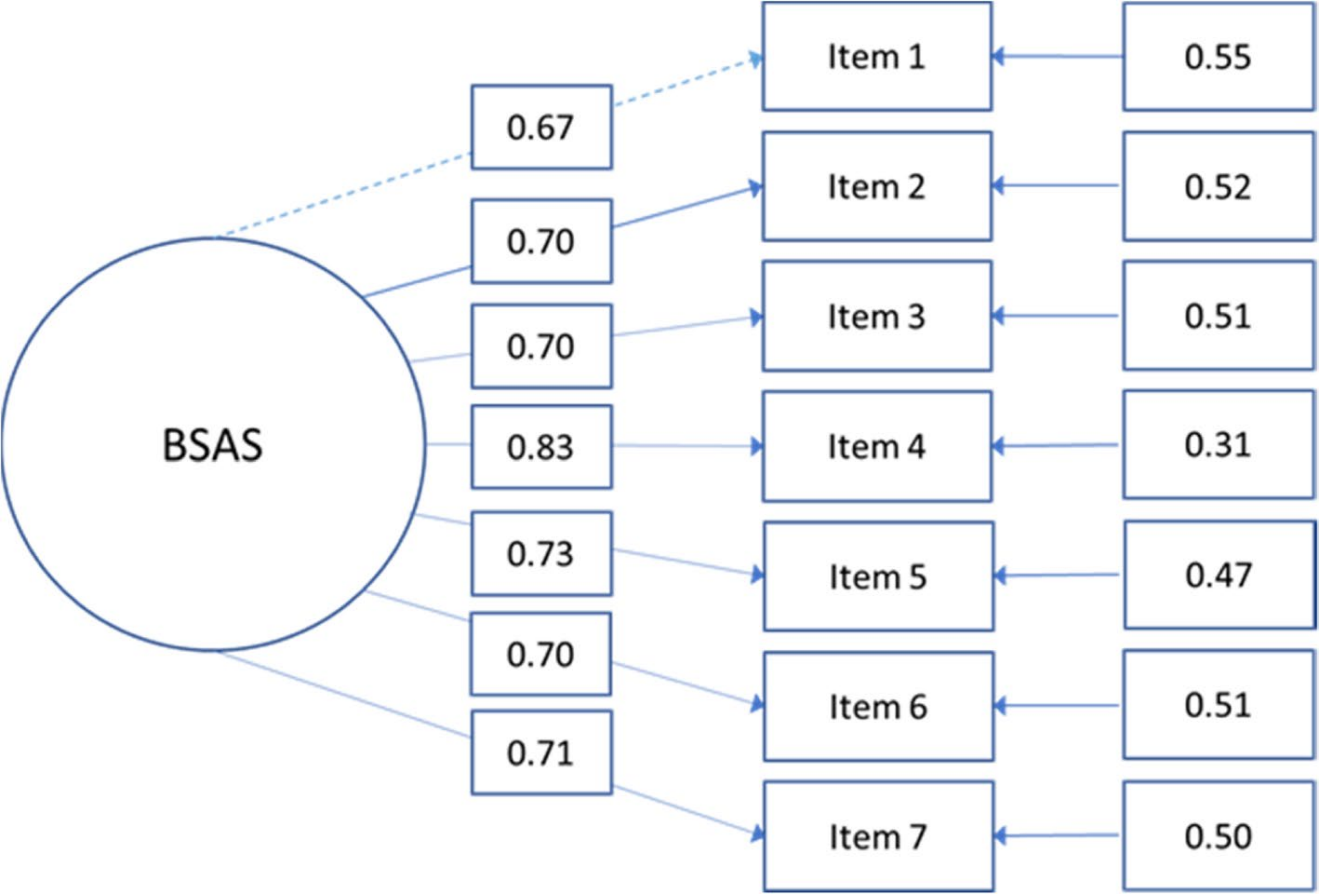
Factorial structure of the Bergen Shopping Addiction Scale (BSAS) showing standardized factor loadings

Missing values showed no discernible pattern (MCAR; Little’s *χ*^2^ = 23.9, *p* = 0.247; Little, 1988), and ranged between 1.50 and 2.60% satisfying the maximum recommended threshold (< 5%; Schafer, 1999). Therefore, IRT assumptions were tested. Firstly, the R Studio-Lavaan package (Rosseel, 2012) was used to fit a confirmatory factor analysis (CFA) and test BSAS unidimensionality employing the weighted least squares means and variance adjusted (WLSMV) estimator due to its ability to deal with polichoric matrices and asymptotic distributions (Enders & Bandalos, 2001). Following the cut-off values outlined in Li (2016), goodness-of-fit indicators suggested sufficient fit to the data (*χ*^2^[14] = 50.39, *p* < 0.001; CFI = 0.989; TLI = 0.984; RMSEA = 0.05 [CI 0.04, 0.07]), with all items loadings saliently on one factor (standardized *λ* = 0.672–0.828; see Fig. 1). Secondly, pairwise residual correlations (LD*χ*^2^ statistics; Chen & Thissen, 1997) showed that items were locally independent (i.e., LD*χ*^2^ < 10; see Supplementary Table 2). Finally, the BSAS showed monotonicity (i.e., raw score continuously increased with increments in *θ*), as demonstrated by the test characteristic curve (TCC).

IRT models were estimated using the Bock-Aitkin marginal maximum likelihood algorithm with expectation–maximization (Bock & Aitkin, 1981). Both the Rasch (*M*_2_[656] = 1569.37; *p* < 0.001; *χ*^2Loglikelihood^ = 12,159.35; RMSEA = 0.04; BIC = 12,303.35; AIC = 12,649.68) and the GRM (*M*_2_[669] = 1831.48; *p* < 0.001; *χ*^2Loglikelihood^ = 12,331.56; RMSEA = 0.04; BIC = 13,256.55) demonstrated sufficient fit (Hu & Bentler, 1999). However, when *α* was constrained to be equal across items, there was as a significant drop in fit (Δ *χ*^2loglikelihood^[7] = 172.21, *p* < 0.01) indicating that the GRM provided superior fit (Gomez et at., 2019). Except for Item 2 (mood modification), all items showed appropriate fit. Therefore, results should be interpreted with caution (see *S*-*χ*^2^ diagnostic statistics in Supplementary Table 3).

### Item Parameters and DIF Statistics

**Fig. 2:**
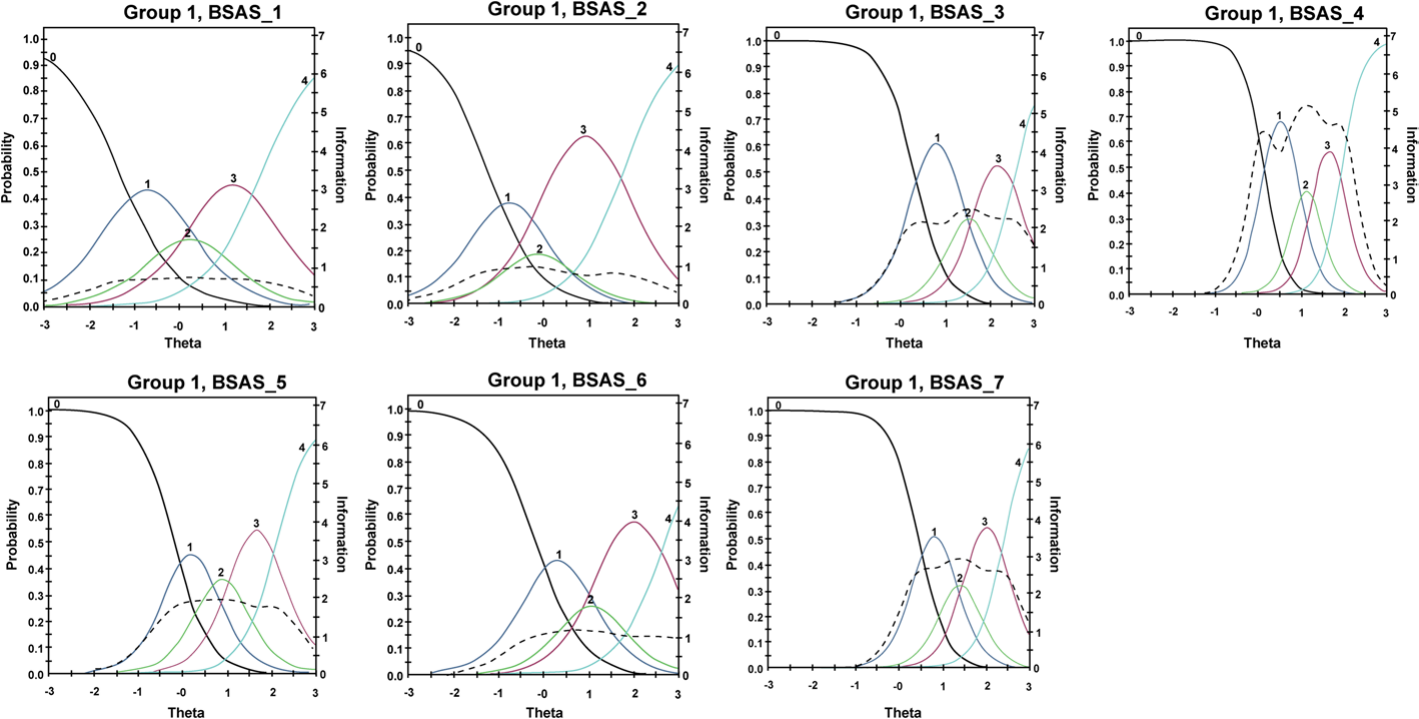
BSAS item characteristic curves (ICCs) and item information functions (IIFs). Here, *theta* (*θ*) represents latent trait levels, and *probability* indicates the likelihood of endorsing an item at different Likert categories. For example, 0 represents *strongly disagree* and 4 represents *strongly agree*. The dotted lines represent conditional reliability indices, with increased levels of information obtained as standard error measurement decreases

Considering *α*, all items were in the high to very high range (0 = non-discriminative; 0.01–0.34 = very low; 0.35–0.64 = low; 0.65–1.34 = moderate; 1.35–1.69 = high; > 1.70 = very high; Baker, 2001). The descending sequence of the items’ *α* is Item 4 (*tolerance*), Item 7 (*presenting problems*), Item 3 (*conflict*), Item 5 (*relapse*), Item 6 (*withdrawal*), Item 2 (*mood modification*), and Item 1 (*salience*; see Table 2 and Fig. 2). Considering *β*, there were fluctuations between the different thresholds across the seven items. For example, while the ascending item sequence of *β* for the first threshold (*β*_1_—*strongly disagree*) was Items 1, 2, 6, 5, 4, 3, and 7, the ascending sequence in the fourth threshold (*β*_4_—*strongly agree*) was Items 2, 1, 4, 5, 7, 3, and 6. Nonetheless, *β* values gradually increased for all items as the “difficulty” of endorsing an item increased, indicating that all items performed accordingly. Considering *c*, values progressively decreased with increments in Likert categories (i.e., from *c*_1_—*strongly disagree* to *c*_4_—*strongly agree*), suggesting that participants’ pseudo-guessing diminished with more “difficult” options.

**Table 2:** Bergen Shopping addiction Scale item discrimination (α), difficulty (β), and pseudo-guessing (*c*) parameters

Item	Label	*α*	*β*_1_	*β*_2_	*β*_3_	*β*_4_		*c*_1_	*c*_2_	*c*_3_	*c*_4_
1	Salience	1.55 (0.17)	− 1.32 (0.16)	− 0.11 (0.10)	0.56 (0.10)	1.83 (0.19)		2.05 (0.20)	0.18 (0.15)	-0.87 (0.16)	-2.83 (0.24)
2	Mood modification	1.77 (0.19)	− 1.25 (0.14)	− 0.34 (0.10)	0.10 (0.09)	1.76 (0.17)		2.20 (0.21)	0.60 (0.17)	-0.17 (0.16)	-3.12 (0.26)
3	Conflict	2.85 (0.33)	0.27 (0.07)	1.27 (0.10)	1.74 (0.14)	2.56 (0.25)		-0.78 (0.23)	-3.62 (0.37)	-4.96 (0.47)	-7.31 (0.75)
4	Tolerance	4.15 (0.53)	0.11 (0.07)	0.92 (0.08)	1.34 (0.10)	1.96 (0.15)		-0.48 (0.30)	-3.82 (0.48)	-5.55 (0.30)	-8.14 (0.94)
5	Relapse	2.15 (0.27)	− 0.16 (0.08)	0.61 (0.08)	1.21 (0.10)	2.17 (0.19)		0.41 (0.20)	-1.54 (0.23)	-3.04 (0.30)	-5.44 (0.49)
6	Withdrawal	1.93 (0.21)	− 0.21 (0.09)	0.75 (0.09)	1.30 (0.12)	2.66 (0.28)		0.40 (0.17)	-1.44 (0.20)	-2.51 (0.24)	-5.15 (0.46)
7	Presenting problems	3.10 (0.38)	0.44 (0.07)	1.17 (0.10)	1.61 (0.13)	2.40 (0.22)		-1.37 (0.27)	-3.64 (0.40)	-4.99 (0.50)	-7.44 (0.78)

*α* represents the capacity of an item to discriminate between varying levels of the behavior (*θ*). *β* indicates the level of behavior needed to endorse an item, where subsequent response rates ‘harder’ than the previous (e.g., *β*_1_ represents *strongly disagree* and *β*_5_ represents *strongly agree*). Standard errors are in parentheses

**Fig. 3:**
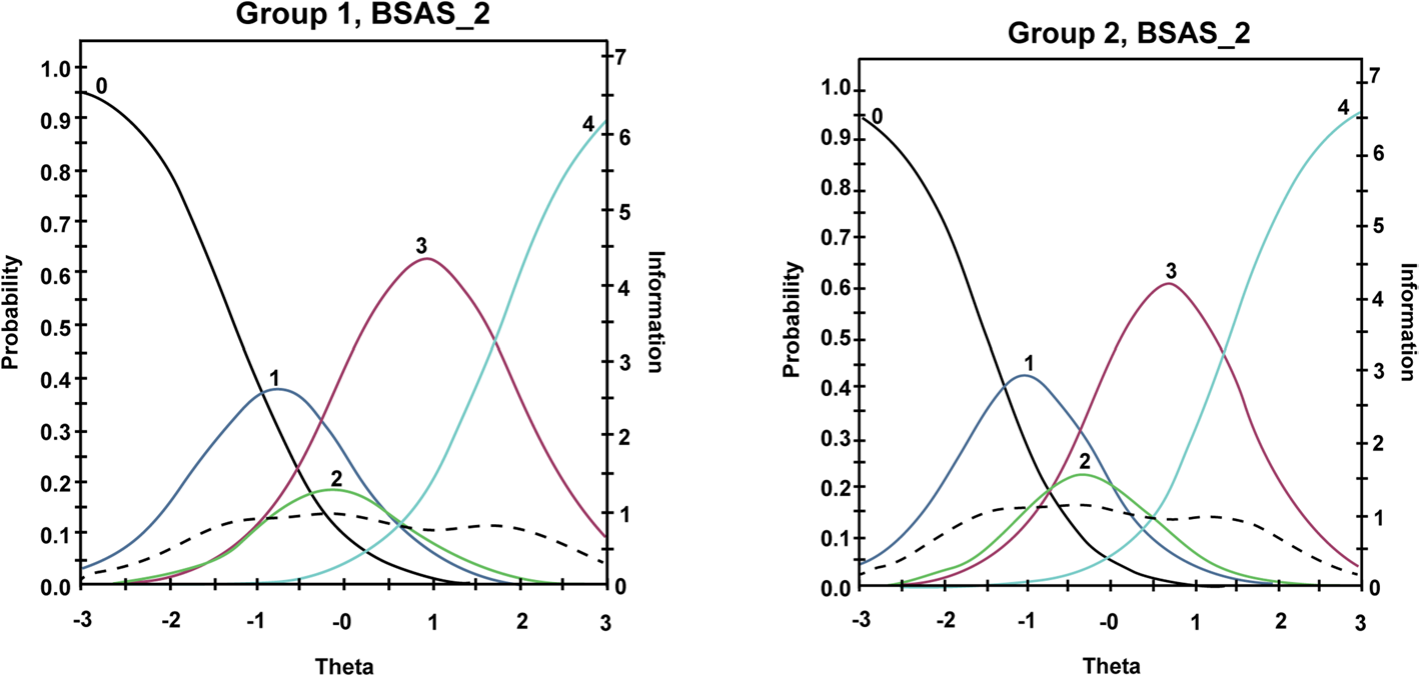
IIF for non-invariant items. Here, Item 2 (mood modification) shows significantly higher *β* for males (group 2) than females (group 1) suggesting that males require higher risk of shopping addiction to endorse this item

Wald tests were employed to identify potentially significant DIF across gender groups. Interestingly, DIF showed that most BSAS items were invariant across gender groups suggesting that the BSAS captures the risk of shopping addiction similarly among males and females. However, Item 2 (*mood modification*) demonstrated non-invariance in *β* across all thresholds (*χ*^2^_cja_[4] = 12.5, *p* = 0.014; Table 3 and Fig. 3). More specifically, *β* threshold for males included *β*_1_ =  − 1.06; *β*_2_ =  − 0.01; *β*_3_ = 0.53; and *β*_4_ = 2.14. Alternatively, *β* threshold for females included *β*_1_ =  − 1.25; *β*_2_ =  − 0.34; *β*_3_ = 0.10; and β_4_ = 1.76. This indicates that males require a higher risk of shopping addiction to endorse this item when compared to female participants.

**Table 3:** Bergen Addiction Shopping Scales differential item functioning (DIF) across male and female participants

Item	Total χ^2^	*df*	*p*	*χ*^2^_a_	*df*	*p*	*χ*^2^_cja_	*df*	*p*
1	7.1	5	0.2116	0.0	1	0.8280	7.1	4	0.1320
2	12.5	5	0.0288	0.0	1	0.9110	12.5	4	0.0142
3	1.1	5	0.9550	0.1	1	0.7922	1.0	4	0.9067
4	4.9	5	0.4242	0.2	1	0.6909	4.8	4	0.3116
5	4.7	5	0.4576	1.0	1	0.3110	3.6	4	0.4567
6	0.5	5	0.9923	0.2	1	0.6533	0.3	4	0.9903
7	3.8	5	0.5770	0.0	1	0.9764	3.8	4	0.4326

While the total *χ*^2^ represents the difference between groups including *α* and *β*, *χ*^2^_a_ represents the difference only including *α*, and *χ*.^2^_cja_ only including *β*

Wald tests using the supplemented expectation–maximization algorithm determined *p* values (significant at .05 level)

Considering item information, interesting fluctuations across items and *θ* levels were observed. More specifically, Item 4 provided the highest level of information between − 0.5SD and + 2.5SD, Item 7 between 0SD and + 2.5SD, Items 3 and 5 between − 0.5SD and + 2.5SD, and Items 1, 2, and 3 provided very limited information across *θ* levels (see Item Information Function, IIF—dotted line, Fig. 1). This indicates that Items 4, 3, 7, and 5 should be prioritized when assessing individuals above mean risk of shopping addiction levels, and more specifically Items 3 and 7 should be emphasized when assessing individuals with extremely high (+ 2SD) risk of shopping addiction scores (see Table 4).

**Table 4:** Item information function (IIF) values for *θ* levels ranging from − 2.8 to 2.8 on the Bergen Addiction Shopping Scale

Item/label	Theta (*θ)* levels														
	− 2.8	− 2.4	− 2.0	− 1.6	− 1.2	− 0.8	− 0.4	− 0.0	0.4	0.8	1.2	1.6	2.0	2.4	2.8
1 Salience	0.20	0.32	0.47	0.60	0.68	0.71	0.73	0.74	0.74	0.72	0.70	0.68	0.62	0.50	0.36
2 Mood modification	0.18	0.32	0.52	0.74	0.88	0.95	0.97	0.94	0.86	0.79	0.79	0.83	0.76	0.58	0.37
3 Conflict	0.00	0.00	0.01	0.04	0.12	0.35	0.90	1.77	2.16	2.07	2.34	2.47	2.31	2.25	1.86
4 Tolerance	0.00	0.00	0.00	0.01	0.07	0.37	1.63	4.13	3.99	4.56	5.12	4.63	4.47	2.06	0.50
5 Relapse	0.01	0.02	0.06	0.16	0.40	0.89	1.49	1.80	1.88	1.93	1.88	1.75	1.73	1.48	0.89
6 Withdrawal	0.02	0.05	0.11	0.22	0.42	0.69	0.95	1.08	1.12	1.15	1.13	1.05	0.99	1.01	0.95
7 Presenting problems	0.00	0.00	0.00	0.02	0.06	0.20	0.62	1.56	2.54	2.70	2.89	2.87	2.63	2.56	1.68
Test information:	1.41	1.72	2.18	2.79	3.63	5.14	8.28	13.03	14.30	14.92	15.85	15.27	14.52	11.45	7.61
Expected standard error	0.84	0.76	0.68	0.60	0.52	0.44	0.35	0.28	0.26	0.26	0.25	0.26	0.26	0.30	0.36

This table shows how information values change at different *θ* levels (risk of shopping addiction)

### IRT Properties at Scale Level and Prevalence

**Fig. 4:**
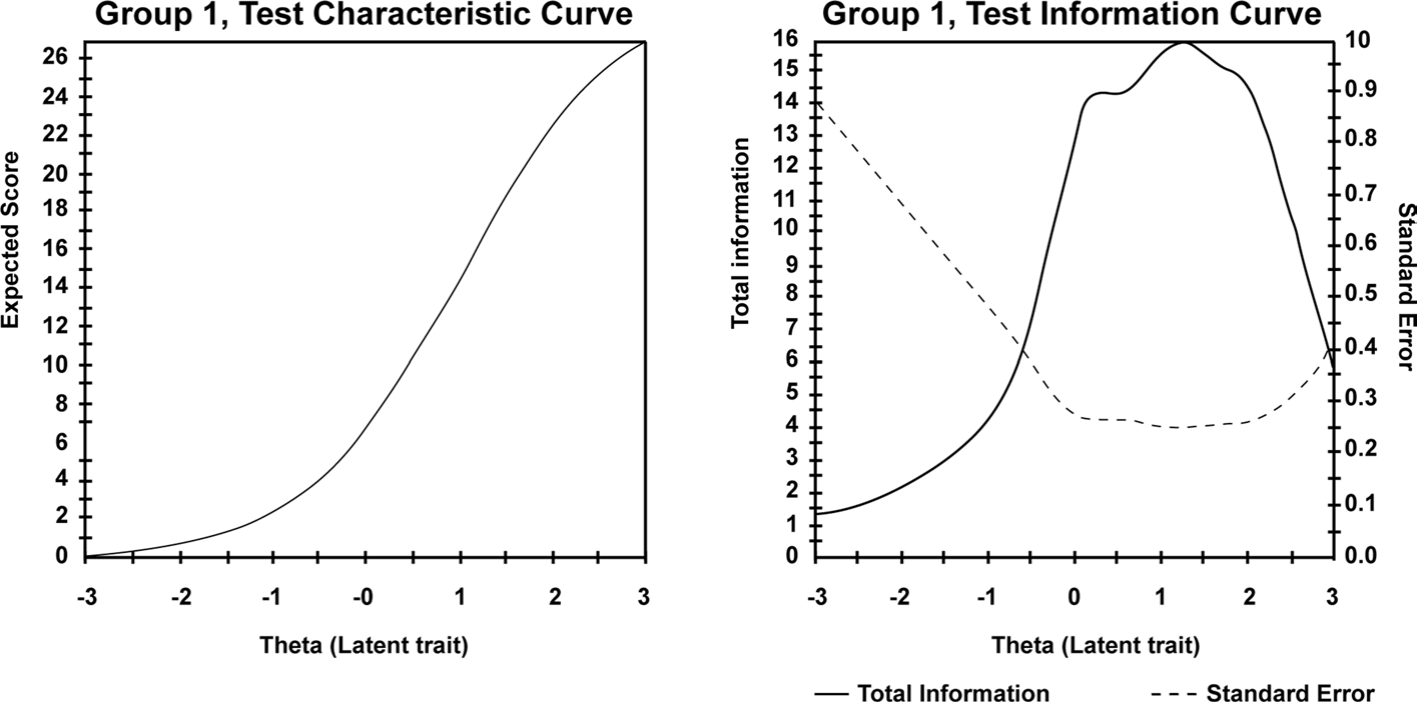
BSAS test characteristic curve (TCC; left panel) and test information function (TIF; right panel). The TCC illustrates the appropriate performance of the Bergen Shopping Addiction Scale as a scale, with risk of shopping addiction increasing as scores increase. The TIF illustrates the conditional effect of standard measurement error (SEM; dotted line) on reliability indices, with increased reliability for reduced SEM

Considering the performance of the scale, the BSAS demonstrated good properties. More specifically, the test characteristic curve (TCC; Fig. 4 left panel) demonstrated a steep increase of BSAS raw scores as *θ* (PSB) increases. Similarly, the test information function (TIF; Fig. 4 right panel) indicated that the BSAS provided increased information for *θ* levels between − 0.5SD and + 2.5SD. However, the scale may not provide such high information at high (+ 2.5SD) and low (− 0.5SD) risk of shopping addiction values.

Considering raw BSAS scores, the SSEAP [*θ*|*x*] identified scaled scores of 5 = 0SD, 14 =  + 1SD, and 23 =  + 2SD based on participants’ responses to all seven BSAS items (Table 5). Therefore, a score of 23 could be recommended as a conditional diagnostic cut-off point (prior to clinical assessment confirmation). Based on this cut-off point for risk of shopping addiction, 8% of participants (*n* = 75) in the sample exceeded it with no significant differences between males and females (*χ*^2^[1] = 0.289, *p* = 0.519). Additionally, raw BSAS scores between 14 and 23 could be used to identify medium risk of shopping addiction.

**Table 5:** Summed Bergen Addiction Shopping Scale score to scale score conversion based on expected a posteriori distribution

Summed score	EAP[θ|***x***]	SD[***θ***|***x***]	Modelled proportion
0	− 1.389	0.610	0.1295
1	− 0.861	0.481	0.1155
2	− 0.556	0.431	0.0949
3	− 0.364	0.422	0.0838
4	− 0.175	0.383	0.0731
5	0.005	0.346	0.0618
6	0.158	0.323	0.0535
7	0.299	0.301	0.0470
8	0.429	0.283	0.0418
9	0.547	0.270	0.0375
10	0.657	0.261	0.0338
11	0.762	0.255	0.0303
12	0.864	0.251	0.0271
13	0.964	0.247	0.0242
14	1.064	0.245	0.0215
15	1.163	0.243	0.0191
16	1.262	0.242	0.0170
17	1.362	0.241	0.0151
18	1.462	0.241	0.0133
19	1.564	0.241	0.0117
20	1.669	0.243	0.0102
21	1.778	0.245	0.0087
22	1.895	0.248	0.0073
23	2.020	0.253	0.0061
24	2.156	0.259	0.0049
25	2.306	0.268	0.0039
26	2.478	0.284	0.0031
27	2.697	0.320	0.0024
28	3.022	0.402	0.0016

While “summed scores” represent raw BSAS scores, EAP (expected a posteriori; EAP[*θ*|*x*]) scores estimate the mean of the posterior distribution of *θ*, given a patterned response to *x*. A measure of the EAP precision can be observed by the posterior standard deviation (SD[*θ*|*x*]). The modeled proportion represent the population distribution likely to obtain a determined raw score based on EAP[*θ*|*x*]

## Discussion

The present study used IRT to (i) investigate the psychometric properties of the BSAS, (ii) assess its differential functioning across males and females, and (iii) estimate the proposed cut-off score for risk of shopping addiction in an adult English-speaking sample. The results demonstrated the BSAS to be a unidimensional measure for the risk of shopping addiction. All seven items showed sufficient discrimination (*α*), difficulty (*β*), and precision, indicating that the BSAS is a psychometrically sound instrument. Additionally, while six items assessed the risk of shopping addiction among males and females similarly, Item 2 (*mood modification*) required significantly higher latent trait levels in males to endorse the item. Finally, a BSAS score of 23 was identified as a proposed cut-off for risk of shopping addiction, with 8% of participants exceeding it, and no significant differences were observed between male and female prevalence rates.

### BSAS Structure

In line with previous studies, the BSAS demonstrated a unidimensional factorial structure and good psychometric properties (Fig. 1; Andreassen et al., 2015). The seven-item BSAS is based upon the components model of addiction (*salience*, *mood modification*, *tolerance*, *withdrawal*, *conflict*, and *relapse*) with the additional inclusion of “*presenting problems*” (Griffiths, 2005). As such, this instrument represents each component with one item, allowing it to maintain its theoretical basis while providing a practical and succinct instrument (Voss et al., 2013). Given the current debate in the field of behavioral addictions, it is important to employ sound theoretical models with clinically identifiable symptoms to contribute to a cohesive body of empirical evidence supporting the recognition of diagnoses and/or disorders such as PSB.

### IRT Properties and DIF Statistics

Considering the BSAS, the risk of shopping addiction inclined sharply as the total score increased, demonstrating a positive correlation between the BSAS scores and shopping addiction presentation. Thus, the BSAS can be an adequate instrument to measure the risk of shopping addiction among individuals with differing levels of problematic shopping presentation. Moreover, all IRT parameters showed interesting variability across items when considering different levels of shopping addiction risk. In line with previous literature, variations in α indicated that the GRM provided the optimal solution to fit the data (Marmara et al., 2021; Zarate et al., 2021). Much like previous literature investigating behavioral addictions, *tolerance* demonstrated the highest α highlighting the item’s ability to detect subtle changes in the risk of shopping addiction (Gomez et al., 2019; Kircaburun et al., 2020; Primi et al., 2021). For example, Gomez and colleagues (2019) indicated that components relating to *tolerance* often show higher discrimination power concerning disordered gaming. *Tolerance* is characterized by a progressively higher engagement in the problematic behavior over time to derive the same pleasure or satisfaction as originally felt when engaging in the behavior, and it *may* lead to addiction or disordered behaviors (James & Jowza, 2019). Therefore, clinical questions related to *tolerance* may be prioritized.

Considering *β*, all items showed a gradual increase between the first and last point of the Likert scale. However, the sequence of *β* changed depending on the items’ threshold. For example, while *salience* showed the lowest *β*_1_ (*strongly disagree*), *mood modification* showed the lowest *β*_4_ (*strongly agree*). *Mood modification* showed a combined low *α* and *β* compared to other items suggesting that this item may indicate less severe problems and may not accurately detect changes in risk of shopping addiction. In line with the self-medication model of addiction and the Interaction of Person-Affect-Cognition-Execution (I-PACE; Brand et al., 2016), *mood modification* may result in individuals engaging in disordered shopping as a maladaptive coping mechanism and to actively seek physiological stimulation with direct and observable effects on mood state and reducing psychological distress (Kovacs et al., 2022). However, results of the present study suggest that *mood modification* may initially attract individuals to engage in the disordered behavior and may not represent chronic addiction-like symptoms or severe problematic behavior (such as *tolerance*). Moreover, in line with previous behavioral addiction studies (Gomez et al., 2019; Lin et al., 2017; Primi et al., 2021), *withdrawal* showed the highest *β*_4_, indicating that this item could be indicative of severe risk of shopping addiction. Interestingly, *presenting problems* showed both high *α* and *β,* suggesting that this newly added item is useful in detecting different levels of risk of shopping addiction.

In addition, DIF statistics confirmed that Items 1 and 3–7 assess shopping addiction risk in the same way for males and females. However, endorsing *β* thresholds in Item 2 (*mood modification*) required a significantly higher risk of shopping addiction among males. In other words, *mood modification* may indicate a less severe risk of shopping addiction among males suggesting that females may be more prone to engage in shopping activities to modify their mood. While previous research has reported mixed findings concerning PSB prevalence rates across males and females (Maraz et al., 2016), theoretical perspectives suggest that females may be conditioned to engage more frequently with shopping activities to modify their mood or stress levels than males (Dittmar, 2005). However, this assertion should be approached with caution considering that gender constructs are being constantly challenged producing fundamental changes in the social fabric of Western societies (Van Droogenbroeck & Van Hove, 2020).

### Item and Scale Precision

Considerable variations in precision were observed across BSAS items. More specifically, *conflict, tolerance*, and *presenting problems* increased precision between − 0.5SD and + 2.5SD. Conversely, *salience*, *mood modification*, and *withdrawal* demonstrated limited precision across latent trait levels suggesting that they may be less accurate compared to other items. Additionally, none of the items provided sufficient information to reliably identify individuals with significantly low levels of shopping addiction (− 3SD to − 2SD). Indeed, the total information function (TIF, Fig. 4 right panel) demonstrated a significant decrease in precision at the scale level reflecting items’ behavior. Nonetheless, the scale provides excellent precision between − 0.5SD and + 2.5SD, suggesting that the BSAS is an accurate and reliable instrument to capture the risk of shopping addiction within this range.

### Cut-off Scores and Prevalence of Risk of Shopping Addiction

Based on the present sample, raw BSAS scores’ translation into scaled scores indicated that a cut-off of 23 or above (out of 28) represents scores + 2SD above the mean, and thus indicate a high risk of shopping addiction (Embretson & Reise, 2013; Thissen, 1995). Accordingly, following this suggested cut-off score, 8% of participants (*n* = 75) were considered at-risk of shopping addiction. Additionally, respondents recording BSAS raw scores between 14 and 23 are suggested to be at medium risk of shopping addiction (+ 1SD to + 2SD), and less than 14 are less likely to experience the risk of shopping addiction. Prevalence rates showed no significant differences between males and females, suggesting a possible bias effect due to social desirability (Biolcati, 2017).

### Limitations, Further Research, and Conclusion

Despite robust findings, there are several limitations in the present study. Firstly, the findings here may not be generalizable to other cultures or languages given that the sample used in this study only comprised English-speaking participants. Secondly, the convenience sampling used to recruit participants may have attracted individuals from the online community and, therefore, may not represent the larger community. This may explain the BSAS’ limited functionality in extremely low scores below the mean. Thirdly, the self-reporting nature of the scale may have enabled social desirability to operate as a confounding factor attenuating potential differences between males and females (Fisher & Katz, 2000). Fourthly, considering that the recruited sample had a large percentage of male participants, further studies with more balanced samples may be needed to replicate the preliminary findings reported here. These limitations may be addressed in future research. Additionally, it may be interesting to investigate shopping addiction by age, as different age groups are likely to have different propensities to develop such behavior.

Despite these weaknesses, the present study provides further evidence of the seven-item BSAS as a valuable and psychometrically sound instrument for assessing the risk of shopping addiction. Overall, the findings observed here demonstrate meaningful differences in item discrimination, difficulty, and precision, which can be used to assess the risk of shopping addiction. Considering IRT item parameters, *tolerance* (Item 4) appears to be the item with the highest discrimination power, while *mood modification* (Item 2) appears to perform differently across the two genders.

## Data Availability

https://github.com/Daniel28052/BSAS.
